# Using the Normalization Process Theory to qualitatively explore sense-making in implementation of the Enhanced Recovery After Surgery programme: "It's not rocket science"

**DOI:** 10.1371/journal.pone.0195890

**Published:** 2018-04-18

**Authors:** Eileen Sutton, Georgia Herbert, Sorrel Burden, Stephen Lewis, Steve Thomas, Andy Ness, Charlotte Atkinson

**Affiliations:** 1 NIHR Bristol Biomedical Research Centre (Nutrition Theme) at the University Hospitals Bristol NHS Foundation Trust and the University of Bristol, Bristol, United Kingdom; 2 Bristol Dental School, University of Bristol, Lower Maudlin Street, Bristol, United Kingdom; 3 School of Nursing, Midwifery and Social Work, University of Manchester, Oxford Road, Manchester, United Kingdom; 4 Derriford Hospital, Derriford Road, Plymouth, United Kingdom; University of California San Francisco, UNITED STATES

## Abstract

**Introduction:**

The Enhanced Recovery After Surgery programme (ERAS) is an approach to the perioperative care of patients encompassing multiple interventions and involving a wide range of different actors. It can thus be defined as a complex intervention. Despite the strength of the evidence-base in its support, the implementation of ERAS has been slow. This paper specifically explores the utility of Normalization Process Theory (NPT) as a methodological framework to aid exploration of ERAS implementation, with a focus on the core NPT construct *coherence*.

**Methods and materials:**

The study employed qualitative methods guided by NPT. Semi-structured interviews were conducted with twenty-six healthcare professionals working in three specialities (thoracic, colorectal, head and neck) in a UK hospital. Data were analysed using an adapted Framework Approach.

**Results:**

*Coherence*, or sense-making work, was key to successful implementation and demonstrated in the importance of participants believing in ERAS both as an individual and as a team. In order to invest in ERAS individuals needed to be able to differentiate its practices favourably with those enacted pre-implementation (differentiation). Participants also needed to understand their specific tasks and responsibilities (individual specification) and build a shared understanding (communal specification), resolving differences in planning meetings. Belief in the worth of ERAS was often aligned to evidence for its effectiveness or benefit to patients (internalization), so implementing ERAS therefore ‘made sense’. Sense-making work had strong links with aspects of implementation related to other NPT constructs including resource issues such as funding for data collection and feedback (reflexive monitoring: systemization) and failure to replace key staff members (collective action: skill set workability).

**Conclusions:**

NPT was found to be a valuable heuristic device to employ in the exploration of ERAS implementation processes. NPT was useful in facilitating recognition of the importance of *coherence* work to successful implementation. However despite participants’ strong beliefs in the worth of ERAS, it was in translating these beliefs into action that barriers were encountered, highlighting the interconnectedness of NPT constructs and the complicated nature of implementing complex interventions.

## Introduction

The Enhanced Recovery After Surgery programme (ERAS) is an approach to the perioperative care of patients encompassing multiple evidence-based interventions which aim to reduce the body’s stress response to surgery and aid rapid recovery [1]. The use of ERAS has been encouraged in NHS hospitals across the United Kingdom since the early 2000s [2]. The programme spans the patient journey from referral from primary care to postoperative follow-up, incorporating around twenty components including preoperative carbohydrate loading, early oral feeding post-surgery and early mobilization, and involves a wide range of different actors in its implementation (S1 Fig)[3]. ERAS is a complex intervention, which has been defined as: “*a cognitive and behavioural ensemble that involves different material and cognitive practices*, *relations and interactions*” [4 [p4].

Studies have shown that in terms of length of hospital stay and complications ERAS has positive outcomes [5]. For example, evidence for colorectal surgery suggests that enhanced recovery programmes may reduce hospital stays by 0.5–3.5 days compared with conventional care [2]. However despite an apparently strong evidence base in its support, in addition to active promotion by the UK government, the implementation of ERAS has been slow [1]. The reasons behind this apparent tardy pace of engagement are poorly understood. Rather than calling for further Randomised Controlled Trials in the area, a recent rapid synthesis of evidence relating to ERAS programmes highlighted the need for further research on how they are implemented, resourced and experienced in NHS settings [2]. There is currently scant qualitative evidence exploring the processes by which the implementation of ERAS is accomplished and the extent to which it is embedded in practice across a range of specialities. Most evidence is limited to colorectal surgery and has been conducted outside of the UK [6–10]. The main aim of the study therefore was to gain a better understanding of the key barriers and facilitators to implementing an ERAS programme across a range of different surgical specialities. This paper specifically explores the utility of Normalization Process Theory as a methodological framework to aid the exploration of ERAS implementation processes by investigating the perspectives of healthcare professionals and managers working in a UK hospital.

### Normalization Process Theory

Normalization Process Theory (NPT), is a middle range theory used to guide empirical enquiry [11]. It is concerned with the social organization of work (implementation); making practices routine elements of everyday life (embedding); and sustaining embedded practices in their social contexts (integration) [12]. NPT and related theories and models have evolved over time with a number of iterations starting with the Normalization Process Model [NPM], which aimed to explain the operationalization of complex interventions in healthcare settings [12]. Following on from the development of NPT, work continued with attempts to develop a general theory of implementation (extended NPT) focussing on resources and possibilities for actors’ contributions to implementation processes [4]. Ongoing work in this area is concerned with developing a quantitative measurement tool, in the form of a 23 item instrument (NoMAD), based on the NPT, which can be used in evaluations of complex health interventions [13].

The NPT was developed from the NPM to include the way that actors make sense of practices [12] and has four core constructs representing the different kinds of work that these actors do as they work around these practices: ***coherence; cognitive participation***; ***collective action***; and ***reflexive monitoring*** (Table 1) [14].

**Table 1:** Normalization Process Theory.

Construct	Sub-component	Description
**1. *Coherence***	**The sense-making work that people do individually or collectively**	
	1.1 *Differentiation*	How a set of practices and their objects are different from each other
	1.2 *Communal Specification*	Building a shared understanding of the aims, objectives and expected benefits of a set of practices
	1.3 *Individual Specification*	Understanding specific tasks and responsibilities around a set of practices
	1.4 *Internalization*	Understanding the value, benefits and importance of a set of practices
**2. *Cognitive Participation***	**The relational work that people do to build and sustain a community of practices around a new technology or complex intervention**	
	2.1 *Initiation*	Whether or not key participants are working to drive an intervention forward
	2.2 *Enrolment*	The strategies used to engage [buy-in], sustain that engagement and help secure implementation
	2.3 *Legitimation*	Ensuring that other participants believe it is right for them to be involved and that they can make a valid contribution
	2.4 *Activation*	Collectively defining the actions and procedures needed to sustain a practice and to stay involved
**3. *Collective Action***	**The operational work that people do to enact a set of practices**	
	3.1 *Interactional workability*	Interactional work that people do when operationalizing ERAS
	3.2 *Relational Integration*	Knowledge work to build accountability and maintain confidence in a set of practices
	3.3 *Skill set workability*	The allocation work that underpins the division of labour built up around a set of practices
	3.4 *Contextual integration*	Managing a set of practices through allocation of resources, execution of protocols, policies and procedures
**4. *Reflexive Monitoring***	**The appraisal work people do to assess and understand the ways that a new set of practices affect them and others around them**	
	4.1 *Systemization*	Collecting information to determine the effectiveness and utility of an intervention
	4.2 *Communal appraisal*	Participants working together to evaluate the worth of a set of practices
	4.3 *Individual appraisal*	Participants working experientally as individuals to appraise its effects on them and the contexts in which they are set
	4.4 *Reconfiguration*	Redefining procedures or modifying practices

Adapted from:

**NPT Core Constructs** [http://www.normalizationprocess.org/what-is-npt/npt-core constructs/] Accessed 16 Jan 2017

Each of these core constructs are comprised of four further sub-components which explore different elements of implementation in greater detail, for example, the construct coherence, which is concerned with the sense-making work that people do individually and collectively, has sub-components of: **differentiation**–understanding how a set of practices and their objects are different from each other; **communal specification**–how people work together to build a shared understanding of the aims, objectives and expected benefits of a set of practices; **individual specification**–how individuals understand their specific tasks and responsibilities around a set of practices; and **internalization**–understanding the value, benefits and importance of a set of practices (Table 1) [14].

In recent years NPT has successfully been employed as a tool to explore the social processes through which new or modified practices, including complex interventions, are implemented in healthcare settings [15]. A number of studies employing the four core constructs of NPT to explore findings have noted the importance of issues of *coherence* as key to the implementation, embedding and integration of interventions in everyday practice [8, 16, 17], although not all of the authors present their findings across all the four sub-components of *coherence*. One of these papers is concerned with the implementation of an ERAS programme in colorectal surgery in Canadian hospitals, the authors focussing their exploration of the *coherence* construct on staff, whom they describe as ‘local champions’, working in surgery, nursing and anaesthesia and driving the implementation process [6]. Blakeman et al [18] have utilised the four constructs and all their sub-components, whilst Frankx et al [19] discuss *patient differentiation* in terms of patient diagnosis within the *coherence* construct. Sanders et al [20] focus their analysis of the introduction of a new system for treating back pain in general practice on aspects of the *coherence* construct, and describe aspects of both *practical coherence* and *relational coherence*. Ong et al also focus on *coherence* in their study on making sense of national guidelines for osteoarthritis in primary care implementation [21]. Building on these analyses this paper examines our findings focussing on aspects of *coherence*, delineating these across its four sub-components, as these issues were seen to be particularly significant as a starting point for implementation in the context of an ERAS programme. The utility of NPT in analysing implementation processes is also explored.

## Materials and methods

Semi-structured interviews were conducted with twenty-six healthcare professionals and managers working in a range of different roles (Table 2) in a regional teaching hospital, who were involved in the implementation of an ERAS programme. Participants included twenty-one working in three specialities (thoracic, colorectal, head and neck), whilst five participants worked in roles that cut across all these specialities. At the time the interviews were conducted all surgical patients in thoracic and colorectal were enrolled onto the ERAS programme, but in the head and neck speciality ERAS was only implemented for one surgical procedure. Interviews were conducted by experienced qualitative researchers (ES and GH) and took place in private areas or offices on hospital premises, or in private rooms in a university Education and Research Centre. Interviews were audio-recorded on an encrypted digital recorder with participants’ written consent and transcribed verbatim by an approved transcription service. Study identifiers are used to denote participants in all study publications, however participants were advised in the study information sheet that due to the size of the study and the need to provide enough background information to make the results meaningful (e.g. by providing the name of the surgical speciality) there was a possibility that they could be identified. Interviews lasted between 23 and 78 minutes (mean 41) and were facilitated by means of an interview guide developed from a review of relevant literature, with topics for discussion structured around the four main constructs of NPT [14] (S2 Fig). Participants were also given the opportunity to highlight issues allied to their role and experience.

**Table 2:** Health care professionals interviewed.

Speciality	SUR/ANS	NUR/AHP	MAN	Total
**Cross-cutting**		3	2	5
**Thoracic**	4	2	1	7
**Colorectal**	2	4	1	7
**Head & Neck**	3	3	1	7
**Total**	**9**	**12**	**5**	**26**

SUR/ANS = Surgeons and Anaesthetists

NUR/AHP = Nurses and Allied Health Professionals [Dietitian, Physiotherapist, Speech & Language Therapist] and Housekeeper

MAN = Clinical Managers (including Ward Managers and Ward Sister) and Trust Management

Participants were recruited using an initial invitation email sent to surgeons already known to the research team. Other healthcare professionals were then recruited by snowball-sampling as each participant was asked to recommend another member of staff who in their opinion had a pertinent role in the ERAS programme. Interviews continued until a range of professionals had been consulted and data saturation had been reached—this is when no new themes were emerging during the interviews. Regular meetings of qualitative team members were held to ensure reflexivity in research and analysis processes, data saturation was discussed at these meetings. Ethical approval for the study was gained from the University of Bristol, Faculty of Medicine and Dentistry Research Ethics Committee (Ref: 121372 (3303)).

Analysis was carried out using an adapted Framework Approach [22] with the aid of the NVivo (Version 10) software package. An initial coding framework was developed that was structured around the four core constructs of NPT and their sub-components [14]. Transcripts were independently coded by both ES and GH who conferred regularly to expand and revise the coding framework in the light of data collection, thereby ensuring robustness in the analysis process. Descriptive accounts of the core constructs, and the sub-components within these constructs, were then developed. Data were compared and contrasted both across and within specialities and staff roles.

## Results

### Coherence: Making sense of ERAS

Within NPT the possibility of the embedding (or normalization) of a practice is seen to depend on: “*a set of ideas about its meaning*, *uses and utility and socially defined competencies*” [11] [p542] which hold it together and make it possible to share and enact it. In this section we therefore explore interviewees’ perceptions of ERAS across the four sub-components of the *coherence* construct, as these are seen to be key to the possibilities for normalization. We then briefly consider how this sense-making work impacts upon and interacts with implementation work across other NPT constructs.

### Differentiation: Distinguishing ERAS from previous practice

In order to invest in ERAS individuals needed to be able to differentiate its practices favourably with those enacted pre-implementation. This required *coherence* work in understanding the potential patient benefits allied to its introduction. Participants provided divergent accounts when they compared ERAS to previous practice. A number of participants asserted that the introduction of ERAS had brought about considerable changes to their day-to-day practice. These changes included positive adjustments in the management of patients and required patients to play a more active role in their own recovery:

I think just by, just by putting enhanced recovery into practice I’d say pain management has changed, and the fact that we do feed patients when they come back now, and that we do give them the *Fortisip* drinks three times a day until they leave … early mobility… so all of that has been like the major change. (NUR/AHP-TH-18)

What we are trying to do is to empower the patient to be the lead of his own recovery. Because we’re telling you know, this is what you are to expect but this is what you are to ask for. So if you are supposed to go out of bed on day one, and if it doesn’t happen, can you ask the nurses to help you get out? (SUR/ANS-TH-14)

ERAS was also described supportively as a “*vessel for change*” (NUR/AHP-CC-4) by one participant who provided the example of ERAS having facilitated the introduction of routine nutrition screening of patients, a practice which was not previously systematized. However some interviewees indicated that rather than requiring major change ERAS had just formalised practice that was already being enacted before the programme’s introduction, by means of the development and introduction of ERAS protocols to direct this practice:

I don’t think there was too much change, it’s more about formalising it all.But, er, in terms of changing practice I don’t think it really has particularly changed. (SUR/ANS-HN-8)

These views may have been linked to participants’ particular roles within a team or ERAS in general, or the specialism in which they were working.

As a set of practices, ERAS held together well enough so it could be operationalized [11], although how it was enacted varied considerably across the different specialisms, for example, in head and neck surgery ERAS had been introduced solely for one surgical procedure, thus impacting on the level of embedding:

Yeah, just a different piece of paperwork that you have to remember to get out … I think it’s just getting used to using it. It’s fine if you implement something new and you’re using it all the time, it just becomes the norm. But if you’re not doing this for every patient you have to try and … remember. (MAN-HN-11)

The small number of patients enrolled onto ERAS in this speciality was found to affect some staff’s ability to differentiate who was an ‘ERAS patient’ and thus enact the relevant care pathway. In contrast the majority of participants working in thoracic and colorectal specialisms—where a blanket, rather than segmental approach to implementation had been adopted—indicated that for them ERAS could be regarded as normal practice:

It’s now become a standard of care so it’s difficult to call it ERAS because it’s standard of care. (SUR/ANS-TH-15)

I think it's probably become part of our normal practice now … because everybody is, comes on the same pathway. So as far as patient care is concerned, they're all on ERAS, so there is no question about it, who should be or who shouldn't be, I think it's a standard of care for me. (SUR/ANS-CO-21)

### Communal specification: Building a shared understanding of ERAS

When participants talked about their role within ERAS they often use the term ‘we’ or described ERAS as a ‘team approach’ thus highlighting their understanding of the significance of good teamwork in its practices:

So it is a team approach to ensure that they’ve got the pathway in place. So it’s not just me. I would be prompting, but there’s lots of people involved in ensuring the patients are on the pathway. (MAN-HN-11)

Speciality specific planning or steering group meetings, where differences about what constituted ERAS practices were resolved and protocols and care pathways developed by staff in different roles, were viewed by a number of participants as key to building this shared understanding:

I think the, the biggest benefit that I had from it, erm, was actually the planning meetings. I think everybody in the same room, erm, talking about things like the tracheostomy protocol and getting agreement, erm, about what we felt was as a team was the right way to do things. (NUR/AHP-HN-9)

Participants revealed that they had received very little ERAS-specific training: a few reported organising some “on the ward” training for nursing staff, but information was generally cascaded down after staff had attended conferences or seminars.

Linked to the sub-construct of *internalization*, it was important that team members had a belief in the aims, objectives and expected benefits of working together as a team to provide evidenced-based care to patients:

There is a good understanding of what is good treatment for our patient … so there is a decision-making that happens as evidence-based as possible. And then it is a question of implementing it. So there is a thinking process. We identify what we think is best with the best evidence we have available, clarify it in the protocol and then implement it. (SUR/ANS-TH-14)

Once protocols and care pathways were established it was important for ERAS to be kept visible so that teams held onto their beliefs in its legitimacy. This might be achieved by the sharing of audit data, for example on reduced length of hospital stay or reduced complications for patients, to be fed back to team members to encourage them to persist with implementation work:

I think a lot of the wards very much appreciate and see the benefits of everything as part of the enhanced recovery. I think sometimes you forget what those benefits are, and I think having it reiterated every now and then really makes a difference. Just to kind of get that motivational push as to why you need to put that effort in and why you need to get your patients up and out of bed. (NUR/AHP-CC-3)

However resource issues had meant that when funding streams attached to ERAS implementation—such as Commissioning for Quality and Innovation payments (CQUINs) that facilitated data collection by dedicated staff—came to an end, teams were no longer provided with such feedback, thus threatening the long term adoption (or success) of ERAS, defined within NPT as embedding and integration.

### Individual specification: Understanding specific roles within ERAS

Coherence work also requires that actors understand their specific tasks and responsibilities surrounding a set of practices [14] along the implementation pathway. Some participants emphasised the importance of their particular role, for example, in providing nutrition support, as part of the ERAS programme as a whole, and consequently to getting ERAS ‘right’:

Nutrition is such a key component, it is an area–those areas that if you get that aspect very right, it can … really make a difference to how successful that enhanced recovery programme is. (NUR/AHP-CC-4)

In some cases this role was as a specific ‘cheerleader’ or ‘champion’ driving implementation processes forward, or as an ERAS ‘enforcer’ consolidating implementation when the programme was in place:

So my role was to, erm, make sure that we bring that cultural change to get people on board, erm, both nursing staff and the medical staff, erm, to develop the pathway which we're using now. (SUR/ANS-CO-21)

So my role is pivotal in enforcing enhanced recovery, that’s why we are very key stakeholders … the technical team, because every day we go on a ward round and … check their [patient’s] progress. (NUR/AHP-HN-12)

Nevertheless there were some participants who were less clear about how their own work fitted within the ERAS programme as a whole, and who felt less engaged (or integrated) as a result:

I think the, erm, team that were trying to bring it in … had a bit more of a challenge to introduce that concept, and which really, then, impacted on the pre-op team, because they didn't really then know where we were going with it, or if we were going with it …or what part we had to play in it. (NUR/AHP-CC-5)

I was involved in the planning and all the paperwork for it, but I don’t really understand why, we were chosen … to be part of the ERAS recovery programme. (NUR/AHP-HN-9)

For other participants ERAS protocol and care pathway development processes had helped to delineate practices related to different ERAS components and staff roles in a way that made sense to them, thus enabling practice:

I mean, the pathway is good … it helps you see what you’re trying to do, especially with the mobility. I think that’s the main bit, and the diary and then planning discharge earlier on. (SUR/ANS-HN-7)

What I think it has made a huge impact on is it has empowered ward nurses to make decisions. (NUR/AHP-CO-24)

### Internalization: ‘Believing in ERAS’

Implementation involves individual knowledge work in assessing the value, benefits and importance of ERAS, linking it to personal norms and values, defined within NPT as *internalization* [4]. As noted above, participants in this study usually referred to their own work in the context of their role within a team, but the majority also conveyed their personal beliefs in the intrinsic value of ERAS in improving patient experience and conferring patient benefit. These beliefs were linked to their commitment to enacting ERAS practice and the sustainability of the programme over time:

I think people have to have … a belief in enhanced recovery and that they want to participate in it and it is the right thing … and I think on the whole the, everybody, the teams that I work with know it’s the right thing to do and it provides a quality service. (MAN-CC-1)

This sounds really corny, but I’m really enthusiastic … about my department and the NHS as a whole. And anything that improves it, the patient’s outcome, and I believe in it, I’m very committed to. (NUR/AHP-CC-5)

Belief in the worth of ERAS was often aligned by participants to existing evidence of its effectiveness or benefit to patients and thus ‘making sense’:

From a medical point of view, if you look at all the … literature from colorectal, er, scientific papers, there has been enough work done to prove that it improves patients' short-term outcomes. (SUR/ANS-CO-21)

We’ve seen it elsewhere and it seems to be a good thing to do, it seems to make sense. It’s not rocket science. (SUR/ANS-HN-8)

Processes of developing protocols within ERAS were understood by a number of participants across the different specialities as constructing an evidence-based tool to aid patient recovery, but conversely the introduction of new protocols was viewed by some staff as an exercise of “*ticking the boxes*” (NUR/AHP-HN-10) that had not improved patient care:

It’s difficult to say now whether it’s [ERAS] going to continue, erm because from some perspectives it’s just another document to fill in, it’s not actually making an impact to patient care. Especially for head and neck patients because we’ve always done certain, certain things, and I don’t, don’t know if it’s made an impact in it. (NUR/AHP-HN-12)

This may have been because they believed that they were already providing good quality patient care pre-ERAS.

The perceived evidence base in support of ERAS was reported by some as having been used constructively to challenge “*entrenched surgical dogmas*” (SUR/ANS-HN-6) surrounding early feeding practices, when contrasting opinions had resulted in tensions in the early stages of implementation. However discussions within steering group meetings and the work of key staff championing ERAS had enabled teams to overcome these tensions.

### Linking coherence across NPT core constructs: Sense-making in action?

*Coherence*, or sense-making work, was key to, and had strong links with, aspects of implementation coded across the other NPT constructs during the analysis process. For example, in NPT “*The production and reproduction of coherence in a practice requires that actors collectively invest meaning in it*” [11] [p543]. The development of that shared investment in ERAS hinged on the role of enthusiastic clinicians or nursing staff who had evolved into ‘leads’ or ‘champions’ and secured initial buy-in for the programme (*cognitive participation*: *enrolment*):

I think the strongest factor is, erm, sort of a senior member of the team with a really key interest in that as an area. So for instance in thoracic surgery, I’m not sure about all the consultants, but there’s at least one consultant that is very keen–was very keen—to implement it, and was very keen to push it forward, and very keen to maintain it, as well. (NUR/AHP-CC-4)

The crucial work of these ‘champions’ was seen to have been enacted alongside top-down pressure from the Trust, which some participants believed may have been prompted by the potential cost-savings linked to reduced length of stay and reduced complications. Managerial support for ERAS implementation was provided by the Trust in the guise of a change management programme.

However after initial implementation, maintaining belief in, and enthusiasm for, the programme was found to be challenging. As noted above, in some cases this was due to resource issues (*collective action*: *contextual integration*) such as discontinued funding for data collection and feedback (*reflexive monitoring*: *systemization*), but there were further tests when key members of staff who had initially championed the programme moved on from a team (*collective action*: *skill set workability*) and were not replaced. This left some interviewees doubting the commitment of the Trust to ERAS in the context of wider organizational pressures:

The Trust sometimes … pays lip service to enhanced recovery, whereas its main concerns, which are the concerns of all acute trusts in the NHS, are those of managing the emergency workload that comes through A&E. (SUR/ANS-TH-15)

This example therefore demonstrates the importance of *coherence* in process of implementation, embedding and integration within the organizational context of different specialities across the Trust, but also recognises the significance of the wider policy context to possibilities for successful implementation of the programme.

## Discussion: Sense-making in implementation

This study has found that believing in ERAS both as an individual and as a team was key to the success of implementing the programme. Positive beliefs in the value of the programme were supported by participants’ assertions of a credible evidence base for ERAS. These findings align with previous research, where beliefs in the legitimacy of novel interventions were found to influence implementation in both positive [8, 23], or negative [16] ways. The beliefs of our participants were closely aligned to their perceptions of relational work around *legitimation*, a sub-component of the *cognitive participation* construct. This construct is concerned with ensuring other participants believe that it is right for them to be involved in ERAS, and that they can make a valid contribution to the programme [14].

In terms of differentiating ERAS practices from those enacted pre-implementation, despite the overwhelmingly positive support for ERAS voiced during the interview process for this study, a few participants were less sure that significant changes had ensued as a result of implementation. Rather than heralding significant change, in their opinion, the introduction of ERAS protocols had merely formalised existing practice. In a previous study of implementation of a shared decision-making (SDM) programme healthcare professionals reported that they were “*already doing SDM*” and demonstrated a lack of motivation to change their practice [17]. This is consistent with some aspects of our findings. Authors have also referred to *differentiation* in terms of how patients or services are defined, for example, demonstrating how conflicting views of depression impacted on creating a shared understanding and actively engaging primary care clinicians in a stepped-care approach [19]. Similarly Knowles et al [24], although not utilising the sub-construct of *differentiation*, describe how participants’ differing definitions of mental and physical healthcare impacted negatively upon collaborative care provision for co-morbid depression and physical health problems. Ong et al found that in their study the majority of general practitioners and nurses distinguished a new intervention positively in terms of pro-active management of patients with osteoarthritis [23]. This evidence combined with our findings indicate that differentiating a novel intervention positively from previous practice may be an important facilitator for successful implementation. The approach to implementation in terms of the throughput of patients may also have an impact on whether practices become embedded, as we found that staff working in specialities where a blanket approach to implementation had been adopted–with all patients placed on an ERAS pathway–were more likely to regard ERAS as standard care.

Knowledge work for the individual (*internalization*) within ERAS was difficult to disentangle from aspects of building a shared understanding of the aims, objectives and expected benefits of a set of practices (*communal specification*). This was evidenced in terms of work undertaken by team members to convince staff who may have been unaware, or sceptical, of its potential benefits. As for other aspects of *internalization* there were apparent links in our analysis to properties of the construct *cognitive participation*. Here the role of enthusiastic clinicians or nursing staff who took on the role of ERAS leads or ‘champions’ was found to be key to securing initial buy-in for the programme, in conjunction with top-down pressure and managerial support. Relevant data relating to the work of ‘champions’ was also coded under the *cognitive participation* sub-construct of *enrolment* in this study thus highlighting the close relationship between making sense of, and building a community of practice around, ERAS [14].

In a previous study of ERAS implementation in colorectal surgery departments that focussed upon the experience of ‘champions’, study participants voiced their beliefs in being the logical or natural person to take on their role. This was despite the variability in their prior knowledge or whether they were appointed or volunteered for their position [8]. Similar to our findings the authors noted the importance of a programme’s alignment with a commitment to evidence-based practice, but additionally found a good fit with a particular department’s existing approach to the use of guidelines facilitated staff buy-in [8]. In our study planning or steering group meetings, which provided an opportunity for team members to meet and shape protocols, were seen as key facilitating factors to building shared understandings of ERAS. Similarly Lloyd et al [17] found that giving team members a role in developing SDM interventions was beneficial, but delineated their findings as aspects of the relational work of the construct *cognitive participation*, rather than *communal specification*.

In terms of the wider impact of sense-making work Sanders et al [20] use the term *relational coherence* to explain the lack of success in the implementation of a new system for treating back pain in the UK. The general practitioners in their study believed implementation would not only disrupt their existing practices, but lead to an unacceptable increase in referral costs. The importance of resource provision, in conjunction with managerial support were also found to be key to the initial implementation of ERAS in our study, and for possibilities of embedding and integration in practice in the longer term.

We recognise that the staff who agreed to take part in interviews for our study may have been those more amenable to ERAS practices, although we sampled widely within the Trust and recruited until saturation was reached. Barriers and facilitating factors to implementation across the three specialities in our study are more fully explored elsewhere [25].

### Using NPT to analyse the implementation of an ERAS programme

The NPT was identified as a potentially useful heuristic device to aid the qualitative analysis of ERAS implementation processes for this study as it had previously been used successfully to examine the implementation of complex interventions in healthcare [15]. NPT was employed to inform the development of an interview guide and as an aid when building a framework for analysis of data from interviews. In line with the experience of other researchers, the theory was found to be useful in identifying factors that promoted or inhibited implementation [15]. Connell et al [23] reported however that using the theory alone was insufficient to clearly identify factors affecting implementation and delivery processes. They found it necessary to use NPT in conjunction with additional implementation frameworks (Conceptual Framework for Implementation Fidelity and Consolidated Framework for Implementation Research). Some researchers have described dilemmas when coding data using NPT [19, 23]. Bamford et al [16] suggest linking NPT to behaviour-change theories to increase its practical utility, whilst others do not mention any problems in using NPT [18, 24].

In our study the starting point for analysis was a framework based on the four core NPT constructs and its sub-components, but during the analysis process we expanded the original framework to accommodate data that initially appeared to be a poor fit by developing additional sub-constructs. For example, a new code related to *communal specification* named *tension* was created as a container for data relating to disagreements connected to the treatment of patients in ERAS. As analysis progressed, further codes were created and others merged to help pinpoint specific issues that were important to the overall analysis. Although expanding the framework was useful for organising our thought processes during analysis, for the purposes of organizing the findings of this study, we found that the broad model of four core constructs and four sub-components outlined in Table 1 provided us with a comprehensive and relevant framework which enabled us to report on key aspects of implementation, embedding and integration. Utilizing NPT helped us to drill down to investigate and unpack important elements of coherence, such as differentiation, in detail. Nevertheless despite the particular focus of this paper upon aspects of *coherence* as key to implementation within ERAS we found that it was difficult to unravel certain features of sense-making from the relational, operational and appraisal work represented in wider NPT model. This highlights the interconnectedness of its constructs and also the complicated nature of implementing complex healthcare interventions.

## Conclusions and implications for future research

By exploring the processes by which the implementation of ERAS is accomplished and the extent to which it is embedded and integrated across three surgical specialities in a UK hospital, this study has provided useful evidence, across a range of previously unexplored contexts, to inform future implementation. The findings enhance the currently limited qualitative evidence base, which to date has largely focussed upon implementation in the context of colorectal surgery.

The NPT was found to be a valuable tool to employ in the exploration of processes of implementation and was used to inform the structure of our interview guide and data analysis framework. In particular, utilizing NPT to analyse our findings enabled us to recognise the importance of *coherence* work to successful implementation. Our study participants demonstrated strong beliefs in the worth of ERAS as “*not rocket science*” (SUR/ANS-HN-8), but a self-evidently good intervention to implement. However it was in translating these beliefs into action that barriers were encountered, thus highlighting the importance of the consideration of implementation processes across all four NPT core constructs when exploring barriers and facilitators to embedding and integrating ERAS practice.

To build on the existing knowledge base and provide a more rounded picture of the programme’s integration at a UK level future work in this area could include carrying out longitudinal qualitative studies or building on qualitative findings to develop a survey of implementation and the findings could be contrasted with quantitative data on length of stay and complications. There is also potential for utilising the NoMAD measurement tool [13] to determine the extent of implementation of different ERAS elements and specific problems encountered, conducted across a wider geographical area.

## Supporting information

S1 Fig ERAS key components and actors. (PDF)

S2 Fig Interview guide. (PDF)

## Abbreviations

CQUIN: Commissioning for Quality and Innovation
ERAS: Enhanced Recovery After Surgery
NoMAD: Measure development based on Normalization Process Theory
NPM: Normalization Process Model
NPT: Normalization Process Theory
