# Correction: Using the Normalization Process Theory to qualitatively explore sense-making in implementation of the Enhanced Recovery After Surgery programme: "It's not rocket science"

**DOI:** 10.1371/journal.pone.0197790

**Published:** 2018-05-17

**Authors:** Eileen Sutton, Georgia Herbert, Sorrel Burden, Stephen Lewis, Steve Thomas, Andy Ness, Charlotte Atkinson

In the Author Contributions section, Georgia Herbert (GH) should be listed as one of the persons who contributed to Formal analysis.
