# Supplementary material for: Using the Normalization Process Theory to qualitatively explore sense-making in implementation of the Enhanced Recovery After Surgery programme: "It's not rocket science"
Source: PLoS One. 2018 Apr 18;13(4):e0195890. doi: 10.1371/journal.pone.0195890 (PMC5906013; doi:10.1371/journal.pone.0195890)
Supplement: S1 Fig — (PDF) [file pone.0195890.s001.pdf]

S1 Figure : ERAS key components and actors – adapted from Bissaillon & Hong, 2014 [3]

<table>
<tr><td colspan="3">ACTIVE PATIENT INVOLVEMENT</td></tr>
<tr><td>Pre-operative</td><td>Intra-operative</td><td>Post-operative</td></tr>
<tr><td rowspan="2">Pre-admission counselling
and information
Early discharge planning</td><td>Temperature control</td><td rowspan="3">Early oral nutrition
Early mobilization
Early catheter removal</td></tr>
<tr><td rowspan="2">Minimally invasive surgery if possible</td></tr>
<tr><td>Reduced fasting duration</td></tr>
<tr><td>Carbohydrate loading</td><td>Avoidance of prophylactic nasogastric tubes and drains</td><td>Defined discharge criteria</td></tr>
<tr><td>No/selective bowel preparation</td><td colspan="2">Use of goal directed perioperative fluid therapy</td></tr>
<tr><td>Venous thromboembolism prophylaxis</td><td colspan="2">Use of multi-modal anti-emetic prophylaxis</td></tr>
<tr><td>Antibiotic prophylaxis</td><td colspan="2" rowspan="2">Use of multi-modal pain management</td></tr>
<tr><td>Pre-warming</td></tr>
<tr><td colspan="3">Audit of compliance and outcomes</td></tr>
<tr><td>Nursing staff
Surgeons ↔
Allied Health Professionals</td><td>Surgeons
Anaesthetists ↔
Nursing staff</td><td>Nursing staff
Allied Health Professionals
Housekeeper</td></tr>
<tr><td colspan="3">← Clinical Managers and Trust Managers →

WHOLE TEAM INVOLVEMENT</td></tr>
</table>
