# Supplementary material for: Using the Normalization Process Theory to qualitatively explore sense-making in implementation of the Enhanced Recovery After Surgery programme: "It's not rocket science"
Source: PLoS One. 2018 Apr 18;13(4):e0195890. doi: 10.1371/journal.pone.0195890 (PMC5906013; doi:10.1371/journal.pone.0195890)
Supplement: S2 Fig — (PDF) [file pone.0195890.s002.pdf]

Interview guide

1. Could you tell me a little about your job role?

   (Prompt: for position classification (banding/medical grade), length of service in clinical area)

2. Why did your ward/hospital decide to implement ERAS?

   (Prompt: How did you hear about ERAS?)

3. How was ERAS set-up in your area?

   (Prompt: Who would you say leads the implementation of ERAS, coordinates the activities, makes the decisions? Who else is part of implementing ERAS?)

4. What is your understanding of ERAS?

5. For you, what are the main components of ERAS?

   (*Prompt about nutritional elements [fluid and carbohydrate loading, no prolonged fasting, early oral nutrition, stimulation of gut motility, prevention of nausea and vomiting] and whether they feel they are as important as the other components)*

6. How does ERAS fit with your current work practices on a day to day basis?

   (*Prompt: how nutritional elements specifically fit.)*

7. Have you had any training with regards to ERAS?

   (Prompt: What training? What did they think of the training?)

8. Do you have a protocol or certain procedures that you follow?

   (Prompt: What are the protocols/procedures they follow?)

9. Describe your role in carrying out ERAS with patients/staff?

10. Do you feel equipped to carry out your role within ERAS?

    (Prompt: Does their role depend on the work of others to carry out ERAS?)

11. How would you describe your level of commitment to ERAS?
    (Prompt: Explore why they have that level of commitment)

12. Are there any factors that facilitate following the ERAS protocol or programme?
    (Prompt: What are the things that help?)

13. Are there any barriers or challenges to following the ERAS protocol or programme?
    (Prompt: What are these barriers/challenges? What has been the most challenging thing about implementing and carrying out ERAS? Have you adopted any strategies to overcome any of these?)

14. Do any other programmes impact on following the ERAS protocol in your area?
    (Prompt: What are these programmes and how do they impact?)

15. Is there anything about the patients you treat that effects doing ERAS?

16. How do you evaluate the ERAS programme both on your own and as a team?
    (Prompt: depending upon position classification - Are you involved in any monitoring of how ERAS is going?)

17. Has your delivery of the programme changed over time?
    (Prompt: What prompted this change? What have been the effects of any adaptations?)

18. How sustainable do you think the ERAS activities are in the long term?

19. Is there anything that you would like to mention that you don't think we've covered?

Thank for time and close interview.
